# Supplementary material for: Epidermal galactose spurs chytrid virulence and predicts amphibian colonization
Source: Nat Commun. 2021 Oct 4;12:5788. doi: 10.1038/s41467-021-26127-9 (PMC8490390; doi:10.1038/s41467-021-26127-9)
Supplement: Supplementary file 1 — Supplementary Information [file 41467_2021_26127_MOESM1_ESM.pdf]

Supplementary Information for

Epidermal galactose spurs chytrid virulence and predicts amphibian colonization

Yu Wang[1, *], Elin Verbrugghe[1], Leander Meuris[2], Koen Chiers[3], Moira Kelly[1], Diederik Strubbe[4], Nico Callewaert[2], Frank Pasmans[1, +], An Martel[1, +, *]

[1] Wildlife Health Ghent, Department of Pathology, Bacteriology and Poultry Diseases, Faculty of Veterinary Medicine, Ghent University, 9820 Merelbeke, Belgium.

[2] Center for Medical Biotechnology, Department of Biochemistry and Microbiology, VIB-Ghent University, 9052 Zwijnaarde, Belgium.

[3] Department of Pathology, Bacteriology and Poultry Diseases, Faculty of Veterinary Medicine, Ghent University, 9820 Merelbeke, Belgium.

[4] Terrestrial Ecology Unit, Department of Biology, Faculty of Sciences, Ghent University, 9000 Ghent, Belgium.

[+] Equally contributed to the study

* Corresponding authors: Yu Wang, An Martel; Email**:** Yu.Wang@ugent.be, An.Martel@ugent.be

Supplementary Figures

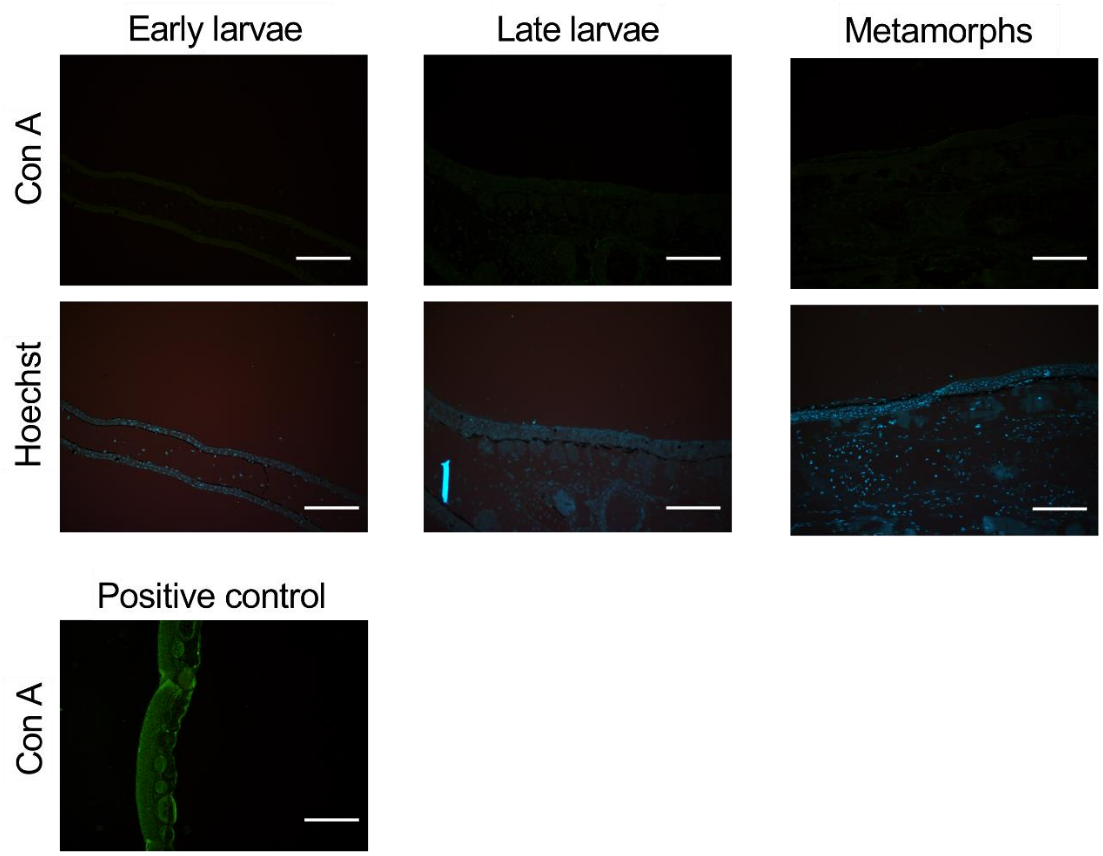

Supplementary Figure 1: Con A and Hoechst staining of different life stages of fire salamander larvae and metamorphs. Skin section of *Alytes obstetricans* was used as a Con A positive control. A representative image is shown (n = 3). Scale bar = 100 µm.

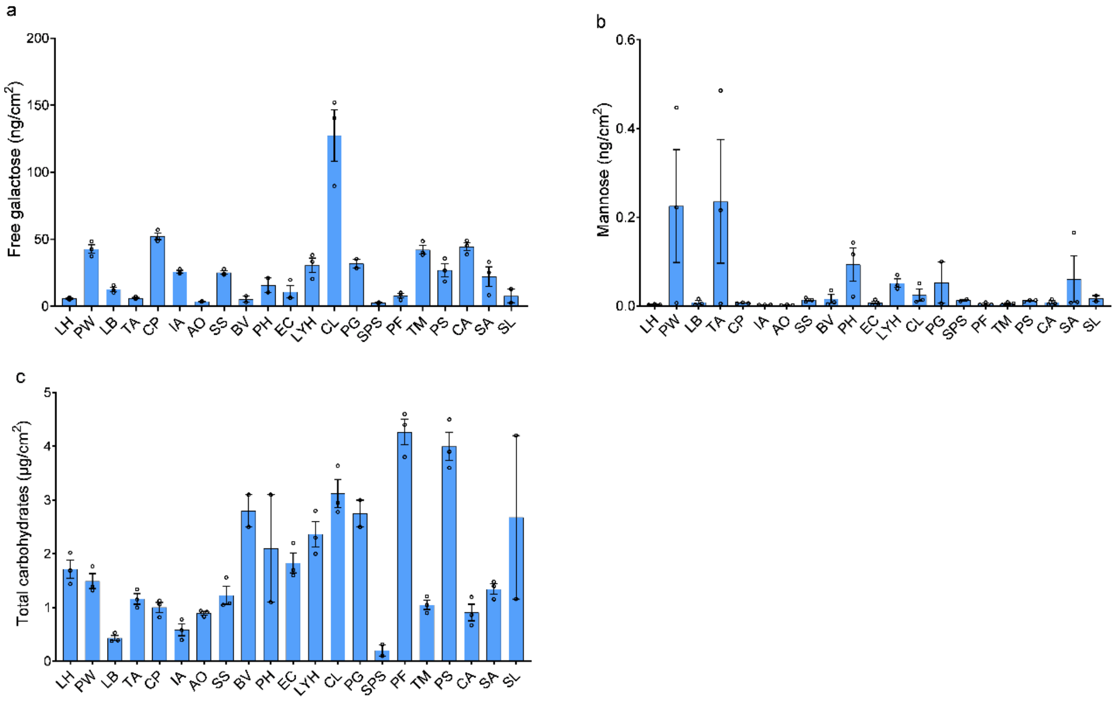

Supplementary Figure 2: Concentration of (a) free galactose and (b) mannose and (c) total carbohydrate per square centimeter of body surface in animal washes. Amphibian species: LH = *Lissotriton helveticus* (*n* = 3), PW = *Pleurodeles waltl* (*n* = 3), LB = *Lissotriton boscai* (*n* = 3), TA = *Triturus anatolicus* (*n* = 3), CP = *Cynops pyrrhogaster* (*n* = 3), IA = *Ichthyosaura alpestris* (*n* = 3), AO = *Alytes obstetricans* (*n* = 3), SS = *Salamandra salamandra* (*n* = 3), BV = *Bombina variegata* (*n* = 2), PH = *Paramesotriton hongkongensis* (*n* = 2), EC = *Epidalea calamita* (*n* = 3), LYH = *Lyciasalamandra helverseni* (*n* = 3), CL = *Chioglossa lusitanica* (*n* = 3), PG = *Plethodon glutinosus* (*n* = 2), SPS = *Speleomantes strinatii* (*n* = 2), PF = *Pelobates fuscus* (*n* = 3), TM = *Triturus marmoratus* (*n* = 3), PS = *Pachyhynobius shangchengensis* (*n* = 3). CA = *Calotriton asper* (*n* = 3), SA = *Salamandra algira* (*n* = 3) and SL = *Salamandra lanzai* (*n* = 2). Data are presented as mean ± SEM; dots represent values of individual animals. Source data are provided as a Source Data file.

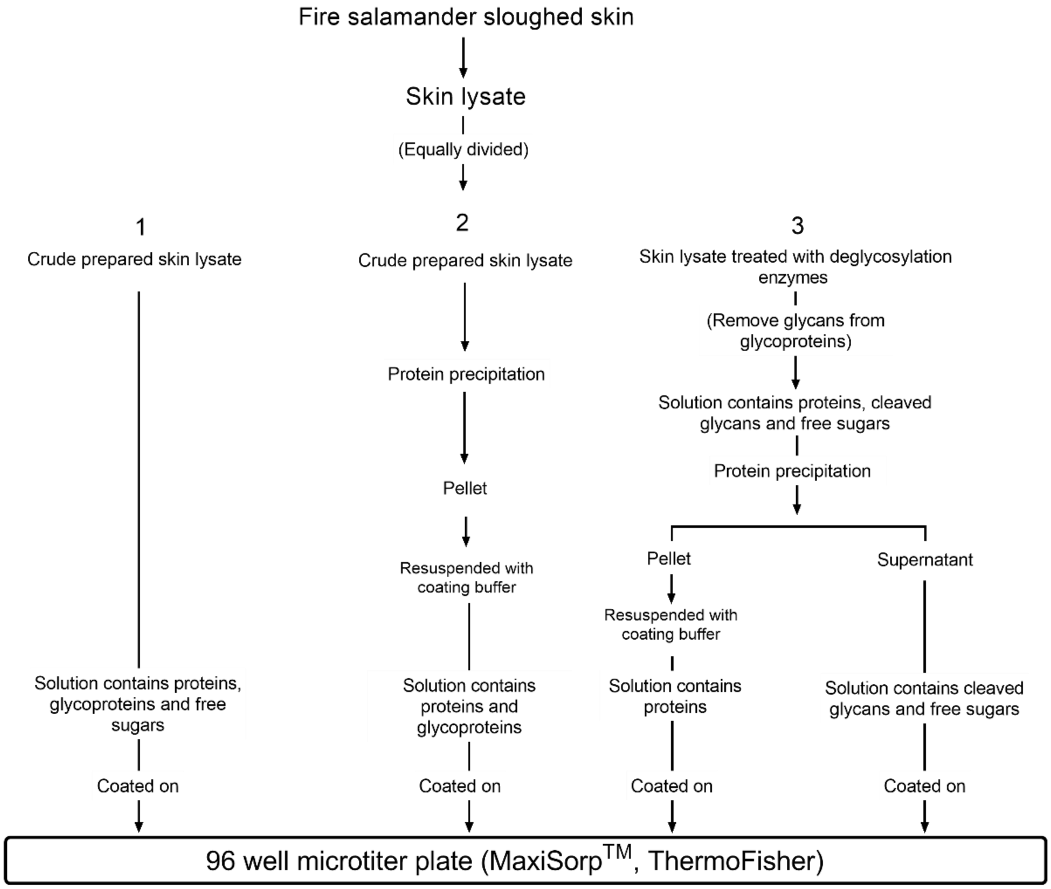

Supplementary Figure 3: Overview of skin lysate binding assay.

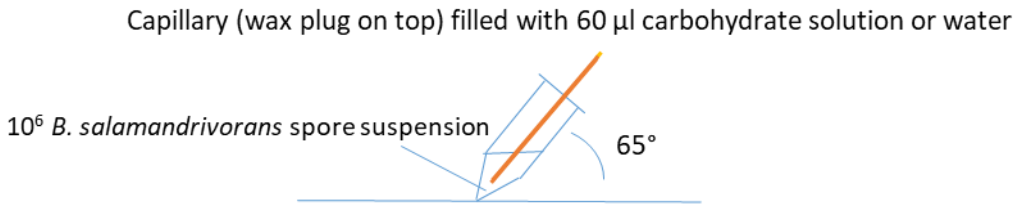

Supplementary Figure 4: Schematic overview of the chemotaxis assay.

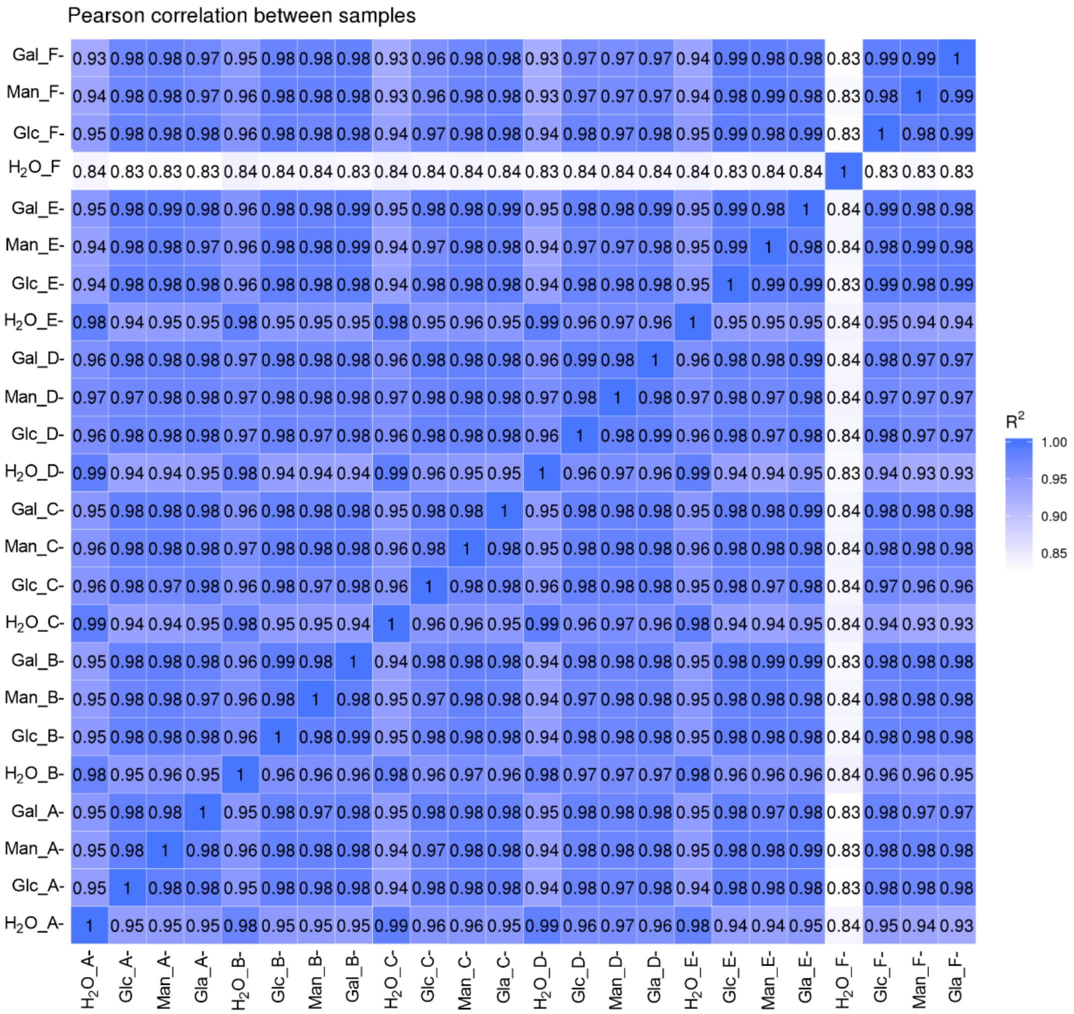

Supplementary Figure 5: Pearson's correlation of transcriptome sequencing between different samples.

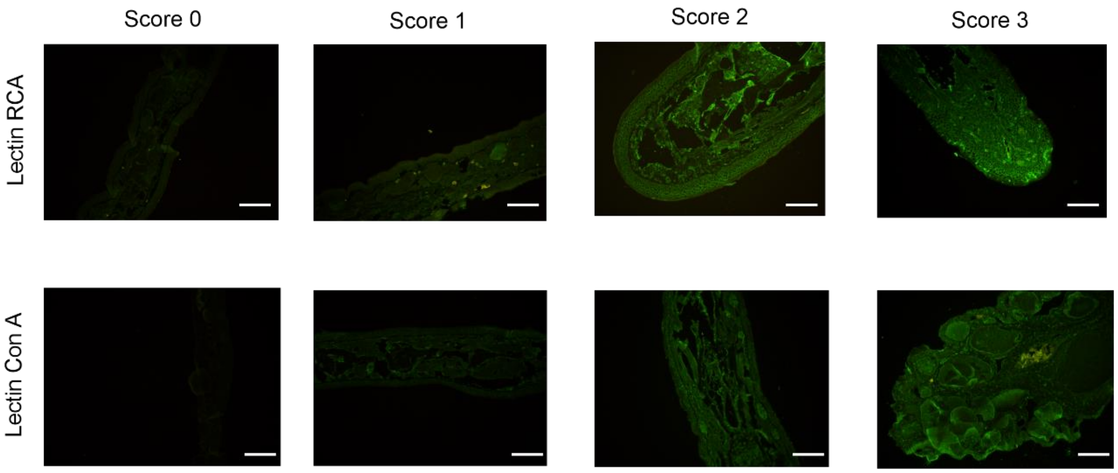

Supplementary Figure 6: Representative pictures for lectin staining evaluation scores. 0 = negative staining, 1 = weak staining, 2 = strong staining, 3 = intense staining. A representative image is shown (n = 3). Scale bar = 100 µm.

Supplementary Tables

Supplementary Table 1: Descriptive statistics accompanying Fig. 1a. Number of *B. salamandrivorans* zoospores bound on microtiter plates coated with different treated skin lysate samples. N = three independent experiments. Mean, standard deviation, Standard Error of the mean, 95% Confidence Interval, minimum and maximum of three independent experiments are given. Negative control: wells coated with only coating buffer. Source data are provided as a Source Data file.

	N	Mean	Std. Deviation	Std. Error of Mean	95% CI for Mean		Minimum	Maximum
					Lower	Upper		
Crude skin lysate	3	2878.38	630.65	364.10	1311.77	4444.99	2163.40	3355.53
Pellet of deglycosylated skin lysate	3	658.89	148.26	85.60	290.60	1027.18	505.87	801.87
Supernatant of deglycosylated skin lysate	3	3950.60	786.81	454.26	1996.06	5905.14	3095.47	4643.93
Pellet of crude skin lysate	3	2751.69	922.44	532.57	460.23	5043.15	1707.60	3456.20
Ammonium sulfate + coating buffer	3	169.07	89.29	51.55	-52.73	390.86	87.20	264.27
Negative control	3	202.09	88.89	51.32	-18.72	422.90	125.87	299.73

Supplementary Table 2: Multiple comparisons based on a (two-sided negative binomial) GLMM linking the number of zoospores attached to the different treatments (see Fig. 1a.) Results present Bonferroni-corrected multiple comparisons test, showing significance of differences in binding of *B. salamandrivorans* zoospores on different treated skin lysate samples. Adjusted *p*-value of $p < 0.05$ is considered as significant. Source data are provided as a Source Data file.

multiple comparisons test	estimate	se	z-value	*P*-Value
Crude skin lysate vs. Pellet of deglycosylated skin lysate	-1.48	0.03	-43.31	<0.001
Crude skin lysate vs. Supernatant of deglycosylated skin lysate	0.32	0.03	9.54	<0.001
Crude skin lysate vs. Pellet of crude skin lysate	-0.07	0.03	-2.13	0.271
Crude skin lysate vs. Ammonium sulfate + coating buffer	2.89	0.04	81.05	<0.001
Crude skin lysate vs. Negative control	-2.70	0.04	-76.32	<0.001
Pellet of deglycosylated skin lysate vs. Supernatant of deglycosylated skin lysate	1.80	0.03	52.78	<0.001
Pellet of deglycosylated skin lysate vs. Pellet of crude skin lysate	0.39	0.03	11.66	<0.001
Pellet of deglycosylated skin lysate vs. Ammonium sulfate + coating buffer	1.42	0.04	39.26	<0.001
Pellet of deglycosylated skin lysate vs. Negative control	1.22	0.04	34.16	<0.001
Supernatant of deglycosylated skin lysate vs. Pellet of crude skin lysate	-1.40	0.03	-41.18	<0.001
Supernatant of deglycosylated skin lysate vs. Ammonium sulfate + coating buffer	3.21	0.04	90.10	<0.001
Supernatant of deglycosylated skin lysate vs. Negative control	3.02	0.04	85.48	<0.001
Pellet of crude skin lysate vs. Ammonium sulfate + coating buffer	2.82	0.04	79.15	<0.001
Pellet of crude skin lysate vs. Negative control	2.62	0.04	74.34	<0.001
Ammonium sulfate + coating buffer vs. Negative control	0.20	0.04	5.29	<0.001

Supplementary Table 3: Descriptive statistics accompanying Fig. 1b. *B. salamandrivorans* zoospore binding to different oligocarbohydrates. N = three independent experiments. Mean, standard deviation, Standard Error of the mean, 95% Confidence Interval, minimum and maximum of three independent experiments are given. Binding normalized to the negative control. GlcNAc = N-Acetylglucosamine, GalNAc = N-Acetylgalactosamine, negative control = wells coated with only coating buffer. Source data are provided as a Source Data file.

	N	Mean	Std. Deviation	Std. Error of Mean	95% CI for Mean		Minimum	Maximum
					Lower	Upper		
GlcNAc	3	208.31	141.24	81.54	-142.54	559.16	86.93	363.33
GalNAc	3	1129.67	441.06	254.65	34.01	2225.33	722.67	1622.73
Mannose	3	1646.87	812.57	469.14	-371.67	3665.41	770.47	2375.27
Lactose	3	1916.98	763.96	441.07	19.20	3814.75	1074.13	2563.87
Negative control	3	182.73	150.19	86.71	-190.36	555.83	66.80	352.40

Supplementary Table 4: Multiple comparisons based on a (two-sided negative binomial) GLMM linking the number of zoospores attached to different oligocarbohydrates (see Fig. 1b.) Results present Bonferroni-corrected multiple comparisons test, showing significance of differences in binding of *B. salamandrivorans* zoospores on different oligocarbohydrates. Adjusted *p*-value of $p < 0.05$ is considered as significant. Source data are provided as a Source Data file.

multiple comparisons test	estimate	se	z-value	*P*-Value
GluNAc - GalNAc	-1.81	0.067	-26.94	<0.001
Lactose - GalNAc	0.51	0.066	7.65	<0.001
Mannose - GalNAc	0.31	0.066	4.72	<0.001
Negative control - GalNAc	-1.99	0.068	-29.37	<0.001
Lactose - GluNAc	2.32	0.067	34.55	<0.001
Mannose - GluNAc	2.13	0.067	31.70	<0.001
Negative control - GluNAc	-0.18	0.068	-2.60	0.071
Mannose - Lactose	-0.19	0.066	-2.93	0.028
Negative control - Lactose	-2.50	0.068	-36.94	<0.001
Negative control - Mannose	-2.30	0.068	-34.12	<0.001

Supplementary Table 5: Multiple comparisons based on a (two-sided negative binomial) GLMM assessing chemotaxis of *B. salamandrivorans* toward free carbohydrates (see Fig. 1c). Results present Bonferroni-corrected multiple comparisons test, showing significance of differences in chemotaxis among different sugars and a water control attractant. Adjusted *p*-value of $p < 0.05$ is considered as significant. Source data are provided as a Source Data file.

multiple comparisons test	estimate	se	z-value	*P*-Value
Glucose - Galactose	-1.67	0.270	-6.174	< 0.001
H20 control - Galactose	-3.57	0.265	-13.462	< 0.001
Lactose - Galactose	-0.73	0.265	-2.751	0.0466
Mannose - Galactose	-1.23	0.266	-4.614	< 0.001
H20 control - Glucose	-1.90	0.286	-6.652	< 0.001
Lactose - Glucose	0.94	0.278	3.375	0.00659
Mannose - Glucose	0.44	0.280	1.560	0.523
Lactose - H20 control	2.84	0.283	10.036	< 0.001
Mannose - H20 control	2.34	0.282	8.308	< 0.001
Mannose - Lactose	-0.50	0.277	-1.802	0.372

Supplementary Table 6: Lectin candidates selected over PFAM annotation pipeline and protein blasting against the FungalDB database. Conserved domains and e-values are listed.

Gene	Domain	e-value
BSLG_00643	Lectin legume-like	1.08E-77
BSLG_00833	Ricin B lectin	6.73E-09
BSLG_03242	Ricin B lectin	1.33E-14
BSLG_05191	Lectin legume-like	6.55E-39
BSLG_02674	Laminin G3 concanavalin A-like lectin	1.34E-05

Supplementary Table 7: Multiple comparisons based on a (two-sided) Linear Mixed Model assessing protease activity detected in supernatants of *B. salamandrivorans* zoospores (see Fig. 3). Results present Bonferroni-corrected multiple comparisons test, showing significance of differences in protease activity. Adjusted *p*-value of $p < 0.05$ is considered as significant. Source data are provided as a Source Data file.

multiple comparisons test	estimate	se	z-value	*P*-Value
Glucose - Galactose	-0.02	0.08	-0.31	0.998
H20 - Galactose	-0.23	0.08	-2.85	0.036
Mannose - Galactose	-0.27	0.08	-3.33	0.008
Protease Inhibitor - Galactose	-1.55	0.08	-19.14	< 0.001
H20 - Glucose	-0.21	0.08	-2.54	0.082
Mannose - Glucose	-0.24	0.08	-3.02	0.021
Protease Inhibitor - Glucose	-1.52	0.08	-18.83	< 0.001
Mannose - H20	-0.04	0.08	-0.48	0.989
Protease Inhibitor - H20	-1.32	0.08	-16.29	< 0.001
Protease Inhibitor - Mannose	-1.28	0.08	-15.81	< 0.001

Supplementary Table 8: Lectin RCA staining patterns in epidermis of ventral skin, dorsal skin, toeclips and tailclips of fire salamanders, alpine newts and palmate newts. Three individuals for each species. SEM = Standard Error of the Mean. Staining scores: 0 = negative; 1 = weak; 2 = strong; 3 = intense. Source data are provided as a Source Data file.

	Fire salamander				Alpine newt				Palmate newt			
	Animal 1	Animal 2	Animal 3	Mean	Animal 1	Animal 2	Animal 3	Mean	Animal 1	Animal 2	Animal 3	Mean
	Score (SEM)	Score (SEM)	Score (SEM)	Score (SEM)	Score (SEM)	Score (SEM)	Score (SEM)	Score (SEM)	Score (SEM)	Score (SEM)	Score (SEM)	Score (SEM)
Ventral skin	3.0 (0.0)	3.0 (0.0)	3.0 (0)	3.0 (0.0)	2.2 (0.1)	2.9 (0.1)	3.0 (0.0)	2.7 (0.3)	0.9 (0.1)	0.3 (0.2)	0.4 (0.1)	0.5 (0.2)
Dorsal skin	3.0 (0.0)	3.0 (0.0)	3.0 (0)	3.0 (0.0)	1.9 (0.1)	2.9 (0.1)	3.0 (0.0)	2.6 (0.3)	0.9 (0.1)	0.4 (0.1)	0.0 (0.0)	0.4 (0.3)
Toeclips	3.0 (0.0)	1.9 (0.1)	2.0 (0)	2.3 (0.3)	3.0 (0.0)	3.0 (0.0)	2.8 (0.1)	2.9 (0.1)	0.9 (0.1)	0.7 (0.2)	0.0 (0.0)	0.5 (0.3)
Tailclips	3.0 (0.0)	3.0 (0.0)	3.0 (0)	3.0 (0.0)	2.4 (0.1)	3.0 (0.0)	3.0 (0.0)	2.8 (0.3)	0.9 (0.1)	0.2 (0.1)	0.4 (0.1)	0.5 (0.3)

Supplementary Table 9: Summary of infection trials, lectin histochemical studies and carbohydrate measurements from animal washes. Order of species, *B. salamandrivorans* susceptibility, infection peak loads, mortality rates, lectin staining scores and percentage of free galactose and mannose are listed. SEM = Standard Error of the Mean. Staining scores: 0 = negative; 1 = weak; 2 = strong; 3 = intense. NA = data not available. Source data are provided as a Source Data file.

Species		*B. salamandrivorans* susceptibility	Infection peak loads (log GE load +1) (SEM)	Mortality rates (%)	References	RCA lectin	Con A lectin	% free galactose	% mannose
Name	Order					Score (SEM)	Score (SEM)		
Salamandra salamandra	Urodela	Susceptible	3.08 (0.2)	100.00	1–4	3.0 (0.0)	1.2 (0.2)	2.10	0.0011
Ichthyosaura alpestris	Urodela	Susceptible	2.90 (0.2)	75.00	2,3	2.7 (0.1)	1.7 (0.1)	4.75	0.0005
Lissotriton helveticus	Urodela	Resistant	0.32 (0.2)	0.00	2	0.6 (0.1)	2.5 (0.4)	0.34	0.0002
Pleurodeles waltl	Urodela	Susceptible	3.05 (0.2)	61.50	1,2	2.1 (0.1)	2.7 (0.3)	2.95	0.0167
Lissotriton boscai	Urodela	Susceptible	4.50 (0.4)	16.67	5	2.4 (0.2)	2.4 (0.2)	2.93	0.0020
Alytes obstetricans	Anura	Resistant	0.44 (0.2)	0.00	2,3	0.4 (0.2)	2.7 (0.3)	0.41	0.0003
Cynops pyrrhogaster	Urodela	Susceptible	3.08 (0.2)	50.00	2	2.0 (0.6)	1.4 (0.4)	5.36	0.0007
Triturus anatolicus	Urodela	Resistant	1.44 (0.8)	0.00	1	0.6 (0.4)	0.8 (0.3)	0.54	0.0196
Triturus marmoratus	Urodela	Susceptible	3.75 (0.4)	100.00	1	1.9 (0.3)	2.2 (0.2)	4.12	0.0006
Calotriton asper	Urodela	Resistant	1.52 (0.9)	0.00	5	3.0 (0.0)	1.9 (0.6)	5.06	0.0009
Salamandra lanzai	Urodela	Not yet determined	NA	NA	NA	3.0 (0.0)	NA	0.59	0.0011
Rana temporaria	Anura	Resistant	0.00 (0.0)	0.00	2	0.3 (0.1)	NA	NA	NA
Bombina variegata	Anura	Tolerant	0.33 (0.3)	0.00	2	0.4 (0.1)	NA	0.19	0.0007
Epidalea calamita	Anura	Resistant	0.54 (0.3)	0.00	2	0.7 (0.1)	NA	0.62	0.0004
Pelobates fuscus	Anura	Resistant	0.00 (0.0)	0.00	2	0.6 (0.2)	NA	0.19	0.0001
Plethodon glutinosus	Urodela	Resistant	0.00 (0.0)	0.00	2	NA	NA	1.18	0.0021
Pachyhynobius shangchengensis	Urodela	Tolerant	0.00 (0.0)	0.00	2	NA	NA	0.67	0.0003
Salamandra algira	Urodela	Not yet determined	NA	NA	NA	NA	NA	1.60	0.0044
Lyciasalamandera helverseni	Urodela	Susceptible	4.32 (0.2)	100.00	5	NA	NA	1.29	0.0023
Chioglossa lusitanica	Urodela	Susceptible	3.37 (0.2)	100.00	5	NA	NA	4.22	0.0008
Speleomantes strinatii	Urodela	Susceptible	3.49 (0.1)	100.00	2	NA	NA	2.55	0.0125
Paramesotriton honkongensis	Urodela	Reservoir	NA	NA	6	NA	NA	1.13	0.0068

Supplementary Table 10: Regression results of RCA scores with *B. salamandrivorans* infection peak loads, mortality rates and percentage of free galactose, using RCA score 0 as the reference group.

Independent variables	Regression Coefficients (β)	t value	*p*-value	95% Confidence interval		R^2	Adjusted R^2
				Lower Bound	Upper Bound		
Dependent variable: infection log (GE load + 1)							
RCA=0 (Constant)	0.257	0.712	0.493	-0.547	1.060	0.872	0.833
RCA=1	0.318	0.667	0.520	-0.745	1.381		
RCA=2	3.338	6.998	0.000	2.275	4.401		
RCA=3	1.973	3.869	0.003	0.837	3.110		
Dependent variable: mortality rates							
RCA=0 (Constant)	5.80E-15	0.000	1.000	-38.510	38.510	0.564	0.434
RCA=1	-7.85E-15	0.000	1.000	-50.944	50.944		
RCA=2	57.043	2.495	0.032	6.099	107.986		
RCA=3	58.333	2.387	0.038	3.872	112.794		
Dependent variable: percentage of free galactose							
RCA=0 (Constant)	0.300	0.316	0.758	-1.814	2.414	0.654	0.550
RCA=1	0.122	0.105	0.918	-2.467	2.712		
RCA=2	3.540	3.046	0.012	0.951	6.129		
RCA=3	2.825	2.431	0.035	0.236	5.414		

Supplementary Table 11: Sequences of the primers and probe used in quantitative real-time PCR (qPCR) for quantifying the number of *B. salamandrivorans* zoospores.

Forward primer (STerF)	5´-TGCTCCATCTCCCCCTCTTCA-3´
Reverse primer (STerR)	5´-TGAACGCACATTGCACTCTAC-3´
Cy5-Probe (SterC)	5´-/Cy5/ACAAGAAAATACTATTGATTCTCAAAC AGG CA/IAbRQSp/-3´
Reference	[4]

Supplementary Table 12: Data quality summary of sequencing results. Glc = glucose; Gal = galactose; Man = mannose.

Sample name	Raw reads	Clean reads	Raw bases	Clean bases	Error rate (%)	Q20 (%)	Q30 (%)	GC content (%)
Glc_B	13345700	12962299	4.0	3.9	0.03	97.83	94.11	48.10
Glc_C	12005909	11740794	3.6	3.5	0.03	97.82	94.05	48.12
Glc_A	12146267	11844741	3.6	3.6	0.02	97.93	94.34	47.94
Glc_F	16948742	16603588	5.1	5.0	0.03	97.46	93.22	48.11
Glc_D	12223887	11955721	3.7	3.6	0.03	97.90	94.18	47.94
Glc_E	17762535	17353477	5.3	5.2	0.03	97.41	93.15	48.31
H_2O_A	14696959	14311212	4.4	4.3	0.02	97.93	94.33	48.05
H_2O_C	14443191	14061285	4.3	4.2	0.03	97.61	93.57	48.24
H_2O_B	11632267	11398588	3.5	3.4	0.03	97.66	93.73	48.12
H_2O_E	14792764	14475716	4.4	4.3	0.03	97.61	93.58	47.97
H_2O_D	16341281	15939571	4.9	4.8	0.03	97.86	94.18	48.13
H_2O_F	15907874	15378637	4.8	4.6	0.03	96.89	92.32	48.61
Gal_B	14343277	14023042	4.3	4.2	0.03	97.82	94.13	48.11
Gal_C	13767498	13304744	4.1	4.0	0.03	97.86	94.10	48.13
Gal_A	13574960	13335481	4.1	4.0	0.03	97.80	94.05	48.02
Gal_F	19623272	19208633	5.9	5.8	0.03	97.63	93.60	48.10
Gal_D	14462516	14218086	4.3	4.3	0.03	97.83	94.08	48.07
Gal_E	18458636	18042658	5.5	5.4	0.03	97.42	93.18	48.30
Man_D	11317621	11056172	3.4	3.3	0.03	97.80	94.01	48.08
Man_E	16480735	16087958	4.9	4.8	0.03	97.39	93.04	48.35
Man_F	17060602	16695412	5.1	5.0	0.03	97.67	93.64	48.25
Man_A	13005267	12741066	3.9	3.8	0.03	97.85	94.13	48.04
Man_B	11788470	11533701	3.5	3.5	0.03	97.48	93.30	48.09
Man_C	13274399	12917376	4.0	3.9	0.02	97.90	94.29	48.15

Supplementary Table 13: Summary of mapping results. Glc = glucose; Gal = galactose; Man = mannose.

Sample name	Total reads	Total mapped reads	Uniquely mapped reads	Multiple mapped reads	Total mapping rate	Uniquely mapping rate	Multiple mapping rate
Glc_B	25924598	21216351	20794533	421818	81.84%	80.21%	1.63%
Glc_C	23481588	19022142	18642392	379750	81.01%	79.39%	1.62%
Glc_A	23689482	19367025	19005977	361048	81.75%	80.23%	1.52%
Glc_F	33207176	28136919	27571043	565876	84.73%	83.03%	1.70%
Glc_D	23911442	19448680	19067074	381606	81.34%	79.74%	1.60%
Glc_E	34706954	29894540	29325493	569047	86.13%	84.49%	1.64%
H_2O_A	28622424	23420602	22948075	472527	81.83%	80.18%	1.65%
H_2O_C	28122570	22791750	22316057	475693	81.04%	79.35%	1.69%
H_2O_B	22797176	18634376	18297507	336869	81.74%	80.26%	1.48%
H_2O_E	28951432	23161442	22721885	439557	80.00%	78.48%	1.52%
H_2O_D	31879142	26315059	25776268	538791	82.55%	80.86%	1.69%
H_2O_F	30757274	23376903	23030764	346139	76.00%	74.88%	1.13%
Gal_B	28046084	23184760	22732566	452194	82.67%	81.05%	1.61%
Gal_C	26609488	21837846	21408651	429195	82.07%	80.45%	1.61%
Gal_A	26670962	22117078	21710534	406544	82.93%	81.40%	1.52%
Gal_F	38417266	32990195	32336144	654051	85.87%	84.17%	1.70%
Gal_D	28436172	23530609	23093569	437040	82.75%	81.21%	1.54%
Gal_E	36085316	30505584	29936126	569458	84.54%	82.96%	1.58%
Man_D	22112344	18201476	17883228	318248	82.31%	80.87%	1.44%
Man_E	32175916	27507455	26972151	535304	85.49%	83.83%	1.66%
Man_F	33390824	28675319	28105689	569630	85.88%	84.17%	1.71%
Man_A	25482132	21089309	20701958	387351	82.76%	81.24%	1.52%
Man_B	23067402	18789259	18445798	343461	81.45%	79.96%	1.49%
Man_C	25834752	20818999	20422249	396750	80.59%	79.05%	1.54%

SI References

1. Martel, A. *et al.* Integral chain management of wildlife diseases. *Conserv. Lett.* **13**, e12707 (2020).
2. Martel, A. *et al.* Recent introduction of a chytrid fungus endangers Western Palearctic salamanders. *Science* **346**, 630 (2014).
3. Stegen, G. *et al.* Drivers of salamander extirpation mediated by Batrachochytrium salamandrivorans. *Nature* **544**, 353–356 (2017).
4. Blooi, M. *et al.* Duplex Real-Time PCR for Rapid Simultaneous Detection of Batrachochytrium dendrobatidis and Batrachochytrium salamandrivorans in Amphibian Samples. *J. Clin. Microbiol.* **51**, 4173 (2013).
5. Bosch, J. *et al.* Batrachochytrium salamandrivorans Threat to the Iberian Urodele Hotspot. *J. Fungi* **7**, 644 (2021).
6. Yuan, Z. *et al.* Widespread occurrence of an emerging fungal pathogen in heavily traded Chinese urodelan species. *Conserv. Lett.* **11**, e12436 (2018).
