# Supplementary material for: Epidermal galactose spurs chytrid virulence and predicts amphibian colonization
Source: Nat Commun. 2021 Oct 4;12:5788. doi: 10.1038/s41467-021-26127-9 (PMC8490390; doi:10.1038/s41467-021-26127-9)
Supplement: Supplementary file 3 — Description of Additional Supplementary Files [file 41467_2021_26127_MOESM3_ESM.docx]

Description of Additional Supplementary Files

Title: Supplementary Data 1

Description: Genes uniquely expressed in galactose-, glucose and Mannose- treated Bsal spores.
