# Supplementary material for: Epidermal galactose spurs chytrid virulence and predicts amphibian colonization
Source: Nat Commun. 2021 Oct 4;12:5788. doi: 10.1038/s41467-021-26127-9 (PMC8490390; doi:10.1038/s41467-021-26127-9)
Supplement: Supplementary file 5 — Reporting Summary [file 41467_2021_26127_MOESM5_ESM.pdf]

nature portfolio

Corresponding author(s): Yu Wang, An Martel

Last updated by author(s): Aug 29, 2021

Reporting Summary

Nature Portfolio wishes to improve the reproducibility of the work that we publish. This form provides structure for consistency and transparency in reporting. For further information on Nature Portfolio policies, see our Editorial Policies and the Editorial Policy Checklist.

Statistics

For all statistical analyses, confirm that the following items are present in the figure legend, table legend, main text, or Methods section.

n/a	Confirmed	
☐	☒	The exact sample size (*n*) for each experimental group/condition, given as a discrete number and unit of measurement
☐	☒	A statement on whether measurements were taken from distinct samples or whether the same sample was measured repeatedly
☐	☒	The statistical test(s) used AND whether they are one- or two-sided<br>*Only common tests should be described solely by name; describe more complex techniques in the Methods section.*
☐	☒	A description of all covariates tested
☐	☒	A description of any assumptions or corrections, such as tests of normality and adjustment for multiple comparisons
☐	☒	A full description of the statistical parameters including central tendency (e.g. means) or other basic estimates (e.g. regression coefficient) AND variation (e.g. standard deviation) or associated estimates of uncertainty (e.g. confidence intervals)
☐	☒	For null hypothesis testing, the test statistic (e.g. *F*, *t*, *r*) with confidence intervals, effect sizes, degrees of freedom and *P* value noted<br>*Give P values as exact values whenever suitable.*
☒	☐	For Bayesian analysis, information on the choice of priors and Markov chain Monte Carlo settings
☒	☐	For hierarchical and complex designs, identification of the appropriate level for tests and full reporting of outcomes
☐	☒	Estimates of effect sizes (e.g. Cohen's *d*, Pearson's *r*), indicating how they were calculated

Our web collection on statistics for biologists contains articles on many of the points above.

Software and code

Policy information about availability of computer code

Data collection	No software was used to collect the data.
Data analysis	Leica Application Suite (LAS) X software was used to take the staining pictures. QPCR results were analysed using the Bio-Rad CFX manager 3.1. The lectin candidate genes were identified with BLASTp (BLAST+2.9.0) and NCBI CDD (v3.16) conserved domain software. For the transcriptome test, we used fastp (version 0.20.0), HISAT2 (version 2.0.5), Featurecounts (version 1.5.0-p3) and R version (version 4.0.0), with R packages DESeq2 (version 1.20.0), clusterProfiler (version 3.8.1), dcGOR (version 1.0.6). Statistical anlysis was performed in R version 4.0.0 and 4.0.3, with packages lme4, multcomp, tidyverse, MASS, VGAM, DHARMa and glmmTMB) or in SPSS (version 26).

For manuscripts utilizing custom algorithms or software that are central to the research but not yet described in published literature, software must be made available to editors and reviewers. We strongly encourage code deposition in a community repository (e.g. GitHub). See the Nature Portfolio guidelines for submitting code & software for further information.

Data

Policy information about availability of data

All manuscripts must include a data availability statement. This statement should provide the following information, where applicable:

- Accession codes, unique identifiers, or web links for publicly available datasets
- A description of any restrictions on data availability
- For clinical datasets or third party data, please ensure that the statement adheres to our policy

All data reported in this study are provided in the Supplementary Information file and Supplementary Data files. Source data are provided with this paper. Carbohydrate binding genes were searched in the NCBI database, under Bioproject PRJNA311566. Reference genome and gene model annotation files used for RNA-seq annotation were downloaded from genome website browser NCBI (https://www.ncbi.nlm.nih.gov/), UCSC (http://genome.ucsc.edu/) and Ensembl

(https://www.ensembl.org/index.html). RNA-seq data are available on the GEO website (http://www.ncbi.nlm.nih.gov/geo/) with Accession number GSE161129 (https://www.ncbi.nlm.nih.gov/geo/query/acc.cgi?acc=GSE161129).

The data was released.

Field-specific reporting

Please select the one below that is the best fit for your research. If you are not sure, read the appropriate sections before making your selection.

- [x] Life sciences
- [ ] Behavioural & social sciences
- [ ] Ecological, evolutionary & environmental sciences

For a reference copy of the document with all sections, see nature.com/documents/nr-reporting-summary-flat.pdf

Life sciences study design

All studies must disclose on these points even when the disclosure is negative.

Sample size

- In vitro tests (skin lysate binding assay- carbohydrate binding assay - carbohydrate chemotaxis test - protease activity test): No sample size calculation was performed. All experiments were conducted in at least triplicate (technical replicates) and all experiments were performed in 3 independent experiments (biological replicates)

- Carbohydrate transcriptome test: The RNAseq analysis was performed in sixfold (6 biological replicates), based on Schurch et al. (2016).

- For mucosome analysis, no sample size calculation was performed. Mucosome samples were collected based on previous results (Greener et al. 2020) in 21 amphibian species in triplicate (also depending on the availability of the amphibian species): Lissotriton helveticus (n = 3), Pleurodeles waltl (n = 3), Lissotriton boscai (n = 3), Triturus anatolicus (n = 3), Triturus marmoratus (n = 3), Cynops pyrrhogaster (n = 3), Ichthyosaura alpestris (n = 3), Salamandra salamandra (n = 3), Lyciasalamandera helverseni (n = 3), Speleomantes strinatii (n = 2), Paramesotriton hongkongensis (n = 2), Plethodon glutinosus (n = 2), Chioglossa lusitanica (n = 3), Pachyhynobius shangchengensis (n = 3), Calotriton asper (n = 3), Salamandra algira (n = 3), Salamandra lanzai (n = 2), Alytes obstetricans (n = 3), Bombina variegata (n = 2), Epidalea calamita (n = 3) and Pelobates fuscus (n = 3).

-Exposure of fire salamander larvae and metamorphs to B. salamandrivorans was based on previous infection experiments (e.g. Stegen et al. 2017, Martel et al. 2013; 2014). (A)Twenty-two early stage and 26 late stage larvae were inoculated with B. salamandrivorans. Ten days after the inoculation all the early stage and sixteen late stage larvae were euthanized. Ten late stage larvae were further kept until five weeks after metamorphosis. (B) Six one week old fire salamander metamorphs were inoculated with B. salamandrivorans and euthanized 10 days after infection.

- For lectin-histochemical staining, no sample size calculation was performed. Skin samples were collected in at least 3 independent animals (depending on the availability of the animals), from the amphibian species Salamandra salamandra (n = 10), Ichthyosaura alpestris (n = 12), Lissotriton helveticus (n = 13), Pleurodeles waltl (n = 11), Lissotriton boscai (n = 3), Alytes obstetricans (n = 10), Cynops pyrrhogaster (n = 3), Triturus anatolicus (n = 3), Triturus marmoratus (n = 3), Calotriton asper (n = 10), Bombina variegata (n = 5), Rana temporaria (n = 10), Epidalea calamita (n = 5), Pelobates fuscus (n = 5) and Salamandra lanzai (n = 3).

Data exclusions

No data were excluded from the analysis.

Replication

- In vitro tests (skin lysate binding assay- carbohydrate binding assay - carbohydrate chemotaxis test) and others: All experiments were conducted in at least triplicate (technical replicates) and all experiments were performed in 3 independent experiments (biological replicates) so that statistical tests that generate P values can then be used to show "statistical significance". All attempts at replication were successful.

- Carbohydrate transcriptome test: 6 biological replicates per condition were included to increase the reproducibility of the experiment. Pearson correlation analysis confirmed the reliability ($R2 > 0.8$), demonstrating the repeatability of the experiment. In addition, the effect on proteases was confirmed in in vitro experiments.

- Protease detection experiment: Three technical replicates were performed per biological replicate, with three biological replicates in total. All attempts at replication were successful.

- Mucosome analysis: free galactose, mannose and total carbohydrates were measured from the animal mucosome of two to three invididuals of each amphibian species. All measures were successful.

- lectin-histochemical stainings: We first collect tail, toe clips, ventral and dorsal skin samples from three individual animals of three amphibian species (fire salamander, alpine news and palmate newts) to investigate the reproducibility of the results in the different body parts. Then we performed lectin-histochemical stainings on tail clips of 15 amphibian species, at least 3 independent animals tested for each species. Staining intensities were scored by three reviewers, respectively scoring the same dataset of pictures blinded three separate times. All stainings confirmed the results.

- In vivo infections: To maximize reproducibility, all amphibians used in experimental trials were captive bred and had no known history of B. salamandrivorans infection. This in-vivo infection trial only performed once with 22 independent fire salamander early stage larvae, 26 independent fire salamander late stage larvae and 6 independent fire salamander metamorphs. All attempted Infection were successful.

Randomization

- In vivo infection experiments: Individuals (depending on the life stage) were randomly assigned.

- Skin lysate binding assay: skin lysate samples were randomly allocated into each experimental condition.
- Carbohydrate chemotaxis test: carbohydrate solutions were randomly allocated and filled in each hematocrit capillaries.
- Carbohydrate binding assay and protease activity test: these tests could not be randomized because different conditions were assessed.
- Carbohydrate transcriptome test: This could not be randomized because different conditions were assessed.
- The suspension of Bsal zoospores were randomly allocated and added into each well of skin lysate binding assay and carbohydrate binding assay, or randomly allocated and added into each condition of carbohydrate chemotaxis test and carbohydrate transcriptome test.
- Mucosome analysis: This could not be randomized because different animals and species were assessed.
- Lectin-histochemical stainings: animals were randomly selected and tissue slides were randomly stained.

Blinding

- In vivo infection experiments: The investigators were not blinded during infection of the animals and data collection as the trial was life stage dependent. All in vivo sample analysis was blinded: The fluorescent intensities were scored by three independent reviewers, respectively scoring the same dataset of pictures blinded three separate times.

- In vitro experiments: The investigators were not blinded to group allocation during data collection or analysis since different conditions needed to be tested and control conditions are always included as a reference during the experimental analysis to consolidate the experimental procedures.

Reporting for specific materials, systems and methods

We require information from authors about some types of materials, experimental systems and methods used in many studies. Here, indicate whether each material, system or method listed is relevant to your study. If you are not sure if a list item applies to your research, read the appropriate section before selecting a response.

Materials & experimental systems

n/a	Involved in the study
☐	☒ Antibodies
☒	☐ Eukaryotic cell lines
☒	☐ Palaeontology and archaeology
☐	☒ Animals and other organisms
☒	☐ Human research participants
☒	☐ Clinical data
☒	☐ Dual use research of concern

Methods

n/a	Involved in the study
☒	☐ ChIP-seq
☒	☐ Flow cytometry
☒	☐ MRI-based neuroimaging

Antibodies

Antibodies used

Fluorescein labeled RCA I (Ricinus Communis Agglutinin I): Vector Laboratories, FL-1081-5
Fluorescein Con A (Concanavalin A): Vector Laboratories, FL-1001-25

Validation

Lectin RCA I binds to β-d-galactose, lectin Con A binds to α-D-mannosyl and α-D-glucosyl (Goldstein and Poretz, 2012). In preliminary tests, the labels were first validated in amphibian species, determining the optimal concentration. Salamandra salamandra skin samples were used as a positive control for Lectin RCA I, Xenopus laevis skin samples were used as a positive control for lectin Con A (Zaccone et al., 1999). For negative controls, lectin RCA I was preincubated with 200 mM galactose and lectin Con A was preincubated with 200 mM mannose + 200 mM glucose, before application to the same skin sections, which resulted in a lack of fluorescent signal.

Animals and other organisms

Policy information about studies involving animals; ARRIVE guidelines recommended for reporting animal research

Laboratory animals

Animals (males/females):

- The mucosome was sampled from adult animals of 21 amphibian species: Lissotriton helveticus, Pleurodeles waltl, Lissotriton boscai, Triturus anatolicus, Triturus marmoratus, Cynops pyrrhogaster, Ichthyosaura alpestris, Salamandra salamandra), Lyciasalamandera helverseni, Speleomantes strinatii, Paramesotriton hongkongensis, Plethodon glutinosus, Chioglossa lusitanica, Pachyhynobius shangchengensis, Calotriton asper, Salamandra algira, Salamandra lanzai, Alytes obstetricans, Bombina variegata, Epidalea calamita and Pelobates fuscus.

-Skin samples were collected from adult animals of amphibian species Ichthyosaura alpestris, Lissotriton helveticus, Pleurodeles waltl, Lissotriton boscai, Alytes obstetricans, Cynops pyrrhogaster, Triturus anatolicus, Triturus marmoratus, Calotriton asper, Bombina variegata, Rana temporaria, Epidalea calamita, Pelobates fuscus and Salamandra lanzai. For species Salamandra salamandra, the skin samples were collected from both larvae and adult animals.

- In vivo infection: (A) Twenty-two early stage and 26 late stage fire salamander larvae were inoculated with B. salamandrivorans. Ten days after the inoculation all the early stage and sixteen late stage larvae were euthanized. Ten late stage larvae were further kept until five weeks after metamorphosis. (B) Six one week old fire salamander metamorphs were inoculated with B. salamandrivorans and euthanized 10 days after infection.

Wild animals	The study did not involve wild animals.
Field-collected samples	The study did not involve samples collected from the field.
Ethics oversight	The animal experiments were performed following the European law and with the approval of the ethical committee of the Faculty of Veterinary Medicine (Ghent University EC) (EC2015/86).

Note that full information on the approval of the study protocol must also be provided in the manuscript.
